# Gastrointestinal flora and gastrointestinal status in children with autism -- comparisons to typical children and correlation with autism severity

**DOI:** 10.1186/1471-230X-11-22

**Published:** 2011-03-16

**Authors:** James B Adams, Leah J Johansen, Linda D Powell, David Quig, Robert A Rubin

**Affiliations:** 1 School for Engineering of Matter, Transport, and Energy, Arizona State University, Tempe, AZ, USA; 2 Doctor's Data, St. Charles, IL, USA; 3 Department of Mathematics, Whittier College, Whittier, CA, USA

## Abstract

**Background:**

Children with autism have often been reported to have gastrointestinal problems that are more frequent and more severe than in children from the general population.

**Methods:**

Gastrointestinal flora and gastrointestinal status were assessed from stool samples of 58 children with Autism Spectrum Disorders (ASD) and 39 healthy typical children of similar ages. Stool testing included bacterial and yeast culture tests, lysozyme, lactoferrin, secretory IgA, elastase, digestion markers, short chain fatty acids (SCFA's), pH, and blood presence. Gastrointestinal symptoms were assessed with a modified six-item GI Severity Index (6-GSI) questionnaire, and autistic symptoms were assessed with the Autism Treatment Evaluation Checklist (ATEC).

**Results:**

Gastrointestinal symptoms (assessed by the 6-GSI) were strongly correlated with the severity of autism (assessed by the ATEC), (r = 0.59, p < 0.001). Children with 6-GSI scores above 3 had much higher ATEC Total scores than those with 6-GSI-scores of 3 or lower (81.5 +/- 28 vs. 49.0 +/- 21, p = 0.00002).

Children with autism had much lower levels of total short chain fatty acids (-27%, p = 0.00002), including lower levels of acetate, proprionate, and valerate; this difference was greater in the children with autism taking probiotics, but also significant in those not taking probiotics. Children with autism had lower levels of species of *Bifidobacter *(-43%, p = 0.002) and higher levels of species of *Lactobacillus *(+100%, p = 0.00002), but similar levels of other bacteria and yeast using standard culture growth-based techniques. Lysozyme was somewhat lower in children with autism (-27%, p = 0.04), possibly associated with probiotic usage. Other markers of digestive function were similar in both groups.

**Conclusions:**

The strong correlation of gastrointestinal symptoms with autism severity indicates that children with more severe autism are likely to have more severe gastrointestinal symptoms and vice versa. It is possible that autism symptoms are exacerbated or even partially due to the underlying gastrointestinal problems. The low level of SCFA's was partly associated with increased probiotic use, and probably partly due to either lower production (less sacchrolytic fermentation by beneficial bacteria and/or lower intake of soluble fiber) and/or greater absorption into the body (due to longer transit time and/or increased gut permeability).

## Background

Individuals with Autism Spectrum Disorders (ASD) often suffer from gastrointestinal problems [1]. The exact percentage suffering from gastrointestinal (GI) problems varies from study to study and depends on the age of the study population, but there is a general consensus that GI problems are common in autism. Population-based studies which do not directly select or bias their samples are the best way to determine the incidence. In a study of 137 children with ASD, 24% had a history of at least one gastrointestinal symptom, with diarrhea being the most prevalent one -- occurring in 17% of individuals [2]. Similarly, a study of 172 children with autism spectrum disorder found 22.7% were positive for GI distress, primarily with diarrhea and constipation [3]. A characterization study of 160 children with ASD found 59% had GI dysfunction with diarrhea or unformed stools, constipation, bloating, and/or gastroesophageal reflux (GERD)[4]. A study of 150 children (50 ASD, 50 controls, and 50 children with other developmental disabilities (DD)) found that 70% of children with ASD presented gastrointestinal symptoms, compared to 28% of typically developing children and 42% of DD children [5]. Additionally, a study by our group of 51 children with ASD compared to 40 typical controls ages 3-15 found that 63% of children with autism were reported to have moderate or severe chronic diarrhea and/or constipation, vs. 2% of the control children [6]. In summary, these studies demonstrate that GI symptoms are common in autism.

GI problems in children with autism may contribute to the severity of the disorder. Abdominal pain, constipation, and/or diarrhea are unpleasant and likely to produce frustration, decreased ability to concentrate on tasks, behavior problems, and possibly aggression and self-abuse, especially in children unable to communicate their discomfort. These problems also result in a decreased ability to learn toilet training, leading to increased frustration for the child and their parents/caregivers.

The cause of these GI problems is unclear, but it appears to partly relate to abnormal gut flora and possibly to the excessive use of oral antibiotics which can alter gut flora. Several studies by our group and others have reported significantly higher oral antibiotic use in children with autism vs. typical children [6-10]. Oral antibiotics were primarily used for treating otitis media (ear infections), which may suggest an impaired immune system. Commonly used oral antibiotics eliminate almost all of the normal gut microbiota, which play an important role in the breakdown of plant polysaccharides, promoting gastrointestinal motility, maintaining water balance, producing some vitamins, and competing against pathogenic bacteria. Loss of normal gut flora can result in the overgrowth of pathogenic flora, which can in turn cause constipation and other problems.

Finegold *et al. *2002 [11] studied fecal samples from 13 children with late-onset autism and 8 controls. They used basic anaerobic culturing techniques to count and isolate microorganisms, followed by Polymerase Chain Reaction (PCR) targeting the 16 S rDNA to identify the isolates cultivated. The number and type of *Clostridium *and *Ruminococcus *species in children with autism differed from the control children. Song *et al. *2004 [12] followed up this study using quantitative real time PCR targeting *Clostridia *strains in stool samples of children with autism and found that *Clostridium *cluster groups I and XI and *Clostridium boltae *had mean cell counts significantly higher than those of control children. Parracho *et al. *2005 [13] used another culture-independent technique, fluorescence in situ hybridization (FISH) targeting *Clostridium *groups, and reported differences in the gut microflora of children with ASD compared to healthy children. In their study, levels of the *Clostridium histolyticum *group of bacteria were higher in the ASD children compared to typical children. *C. histolyticum *bacteria are recognized toxin producers and may contribute to gut dysfunction. Finegold *et al. *2010 [14] used a high throughput sequencing technique, i.e., pyrosequencing to investigate gut bacteria in children with autism vs. controls, and found several differences at the phylum level, including higher levels of *Bacteroidetes *in the severely autistic group, and higher levels of *Firmicutes *in the control group. Additionally *Desulfovibrio *species and *Bacteroides vulgatus *were present in higher numbers in autistic than controls

Treatment studies using a minimally absorbed oral antibiotic (vancomycin) to treat abnormal gut flora showed significant temporary improvements in behavior for children with late-onset autism [15], but the benefits were lost after treatment stopped. This study demonstrated the importance of gastrointestinal flora and the difficulty in permanently normalizing them.

Much focus has been given to the presence and abundance of *Clostridium *groups in the intestines of autistic children. Finegold 2008 [16] hypothesized that 1) the relapse of some autistic kids after antibiotic treatment is caused by the presence of *Clostridium *spores, 2) the incidence of autism is related to the widespread exposure to *Clostridium *spores, and 3) the increase of multiple autism cases within a single family is also related to contact with spores. Finegold also discussed the fact that propionate has been shown to have severe neurological effects in rats [17,18] and *Clostridia *species are propionate producers [19]. No human studies have been conducted to test whether the relative proportion of propionate and/or its absolute concentration correlates to autistic symptoms. However, studies by MacFabe *et al *[17] have demonstrated that injecting propionate directly into specific regions of rat brains *in vivo *can cause significant behavior problems [17,18,20].

There have also been reports of decreased activity of digestive enzymes in children with autism. One study by Horvath and Perman 2002 [21] reported that 44 of 90 (49%) children with autism who underwent endoscopy (because they had significant gastrointestinal problems) had deficiencies in one or more disaccharidase enzymes, especially lactase and maltase. They reported that all of the children with low enzyme activity had loose stools and/or gaseousness.

A recent study of children with autism and their first-degree relatives found that 37% and 21%, respectively, had increased intestinal permeability based on a lactulose/mannitol test, compared to 5% of normal subjects. They also found that autistic patients on a gluten-free, casein-free diet had significantly lower intestinal permeability [22].

In summary, gastrointestinal problems are common in children with ASD and may contribute to ASD behavioral symptoms. However, more research is needed. In this study we investigate some gut flora and biomarkers of GI function in children with ASD compared to typical children. The gut flora investigated include both beneficial and pathogenic bacteria that are easily cultured, but the culture methods used were able to detect only some of the bacteria present, and some anaerobic bacteria such as clostridia were not able to be detected.

## Methods

This paper reports on the study of children with autism compared to typical children. This study was conducted with the approval of the Human Subjects Institutional Review Board of Arizona State University.

Participants: Participants were recruited from Arizona with the help of the Autism Society of America - Greater Phoenix Chapter. All parents and (where possible) children signed parent consent/child assent forms. The control group was also recruited with the help of the Autism Society of America - Greater Phoenix Chapter, but the control group participants were not related to children with autism.

### Enrollment criteria

1) Age 2 1/2 to 18 years

2) No usage of any type of antibiotic or antifungal medications within the last month.

3) Autism Group: diagnosis of autism, PDD/NOS, or Asperger's by a psychiatrist or similar professional (no additional assessment was done in this study)

4) Control Group: In good mental and physical health: no stomach/gut problems such as chronic diarrhea, constipation, gas, heartburn, bloating, etc. No Attention Deficit Disorders (ADD/ADHD). Unrelated to an individual with autism (not a brother, sister, parent, aunt, or uncle). Based on parent report (no additional assessment was done in this study).

### Participants

The characteristics of the study participants are listed in Table 1. They were similar in age, but the control group had a higher percentage of females, and a lower severity of gastrointestinal problems.

**Table 1:** Characterization of participants.

	Autism/Aspergers	Control	*P*-value
Total # participants	58	39	--
Male/Female	50/8	18/21	
Age (years)	6.91 ± 3.4	7.7 ± 4.4	n.s.
% Autism/Aspergers	94.8/5.2	--	--
ATEC	68.8 ± 29.5	--	--
6-GSI	3.9 ± 2.5	1.3 ± 1.4	0.000001^1^
Probiotic Usage	33%	5%	
Seafood Consumption	12% high (> 2x/month)	33% high (> 2x/month)	
	2% low (1-2x/month)	3% low (1-2x/month)	
	53% none	64% none	
	33% unknown		
Fish Oil Consumption (daily)	36%	0%	

^1^The difference in 6-GSI scores is expected since controls were chosen not to have significant GI problems, whereas children with autism were accepted into the study regardless of their GI problems.

### Study Protocol

1) The study was explained to participants and informed parent consent/child assent was received.

2) Parents filled out a questionnaire on their child's gastrointestinal status. Parents of children with autism also filled out the Autism Treatment Evaluation Checklist (ATEC) (Rimland and Edelson 2000) [23].

3) Stool samples were collected and sent by 2-day express shipping to Doctor's Data in a blinded fashion.

### Severity Scales

Autism severity was assessed with the Autism Treatment Evaluation Checklist (ATEC), an instrument which was designed to provide a quantitative assessment of autism severity. It is composed of four subscales: 1) speech/language/communication, 2) sociability, 3) sensory/cognitive awareness, and 4) health/physical behavior. The sum of the scores for each subscale gives the total ATEC score. The internal reliability of the ATEC is very high (0.94 for the Total score), based on a split-half reliability test on over 1,300 completed ATECs.

Gastrointestinal symptoms were assessed using a modified version of the GI Severity Index [24]. Specifically, we included only the first six items (constipation, diarrhea, stool consistency, stool smell, flatulence, and abdominal pain), but did not include "unexplained daytime irritability", "nighttime awakening," or "abdominal tenderness." We call this shortened version the 6-GI Severity Index (6-GSI).

### Lab Methodology

All laboratory measurements were conducted by Doctor's Data (St. Charles, IL, USA - http://www.doctorsdata.com). Doctor's Data is certified by CLIA, the Clinical Laboratory Improvement Amendments program operated by the US Department of Health and Human Services which oversees approximately 200,000 laboratories in the US.

### Collection, Preservation & Transport

A single stool sample was collected, and then material was added to 3 collection tubes:

1. Remel Cary Blair Transport Media Tube was used for the transportation, preservation and examination of stool specimens for enteric bacteria. This tube provides a low nutrient, low oxidation-reduction potential and high pH environment which allows for successful recovery of stool pathogens and other organisms of interest. Internal stability studies by Doctor's Data have shown consistent identification and recovery of organisms measured every 2 days for 14 days after collection.

2. Remel SAF Fixative Tube was used to preserve stool and maintain the morphology of the parasites and cellular material.

3. An empty sterile tube was used for Chemistry testing. Stool was frozen immediately after collection, and sent frozen to the laboratory in an insulated container with a frozen cold pack. All samples were verified as frozen on arrival at Doctor's Data.

### Bacterial/Yeast Culture, ID & Susceptibility

The process of bacterial cultivation involves the use of optimal artificial media and incubation conditions to isolate and identify the bacterial etiologies of an infection as rapidly and as accurately as possible. For this study we used many growth media including 5% Sheep Blood Agar (BAP), MacConkey Agar (MAC), Columbia Agar (CNA), Hektoen Enteric Agar (HEK), GN Broth, Sab/Dex Gent (yeast media), and Modified Columbia Agar.

### Quantification

The quantification of culture-based methods was done on a scale of 1-4, defined as: 1+ = Rare, 2+ = Few, 3+ = Moderate, and 4+ = Many or Heavy growth of microorganisms. The estimates of recovery are:

0 = no growth, less than 10^3 ^colony forming units/gram of feces = 1+ growth

10^3 ^- 10^4 ^colony forming units/gram of feces = 2+ growth

10^5 ^- 10^6 ^colony forming units/gram of feces = 3+ growth

> 10^7 ^colony forming units/gram of feces = 4+ growth

Colony-forming unit (CFU or cfu) is a measure of viable bacterial or fungal numbers. Unlike direct microscopic counts where all cells, dead and living, are counted, CFU measures viable cells.

### Identification

The Vitek^®^2 Gram Negative (GN) identification card was used in conjunction with the Vitek^®^2 system for the automated identification of microorganisms of the family Enterobacteriacaea. In addition, a select group of glucose non-fermenting gram-negative bacteria, *Pasteurella multocida*, and members of the family *Vibrionaceae *can be identified.

The Vitek^®^2 Gram Positive (GP) identification card was used in conjunction with the Vitek^®^2 system for the automated identification of microorganisms of clinically significant streptococci, staphylococci, and a selected group of gram-positive bacilli.

The Vitek^®^2 Gram Negative Antimicrobial Susceptibility Test (GN-AST) card was used in conjunction with the Vitek^®^2 system for the automated quantitative or qualitative susceptibility testing of isolated colonies for most clinically significant aerobic gram-negative bacilli. The system evaluates each organism's growth pattern in the presence and absence of antibiotics. The Minimum Inhibitory Concentration (MIC) is determined based on growth characteristics.

The Vitek^®^2 Yeast (YST) identification card was used in conjunction with the Vitek^®^2 system for the automated identification of most significant yeasts and yeast-like organisms.

### Parasitology

Both a concentration method and a trichrome stain were used to identify parasites. The purpose of the concentration method is to separate parasites from fecal debris and to concentrate any parasites present through sedimentation. The parasitology processing and identification procedure is a two-step process. The concentration process is the first step, which uses centrifugation. Ethyl acetate is used as an extractor of debris and fat from the feces and leaves the parasites at the bottom of the suspension. Wet mounts are examined for parasites from the concentrated sediment using iodine as a stain to enhance morphology.

Stained fecal films are the single most productive means of stool examination for intestinal protozoa. The permanent stained smear facilitates detection and identification of cysts and trophozoites and affords a permanent record of the protozoa encountered. The trichrome technique of Wheatley for fecal specimens is a modification of Gomori's original staining procedure for tissue. It is a rapid, simple procedure that produces uniformly well-stained smears of intestinal protozoa, human cells, yeast cells and artifacts.

The parasitology test was used on the first 20 autism samples only, which were all negative. It was then decided to do no additional testing on other samples.

### Stool Chemistry Testing

**Lysozyme **(muramidase) is a protein that belongs to the group of alkaline glycosidases. The main source for fecal lysozyme is the intestinal granulocytes. To some extent, mononuclear cells in the bowel lumen also secrete lysozyme actively. Lysozyme is an enzyme with bacteriocidal properties. It is secreted by recruited macrophages and monocytes at the site of inflammation. Lysozyme is useful in the diagnosis and monitoring of Crohn's Disease and also in bacterial, viral, allergenic, and autoimmune caused bowel inflammations [25,26]. An Enzyme-Linked-Immuno-Sorbent-Assay (ELISA) was used for the quantitative determination of lysozyme in stool, using the Lysozyme ELISA Kit (ALPCO Diagnostics Cat. No. 30-6900) [27].

### 

**Lactoferrin:** In inflammatory diarrhea, fecal leukocytes are found in the stool in large numbers [28]. Lactoferrin serves as a marker for leukocytes in acute diarrhea. It is very stable and is not degraded during infections by the toxins of pathogens. The purpose of this test is to differentiate between inflammatory bowel disease (IBD) and non-inflammatory bowel syndrome (NIBS) [29-31]. The measurement was performed with IBD-Scan^® ^Kit (TechLab^^®^^Blackburg, VA) [32].

**Secretory IgA (sIgA) **is the major immunoglobulin in saliva, tears, colostrum, nasal mucous, mother's milk, tracheobronchial and gastrointestinal secretions [33,34]. It plays a major role in preventing adherence of microorganisms to mucosal sites, in activating the alternative complement pathway, and in activating inflammatory reactions [35]. Fecal sIgA is elevated in a response of the mucosa immune system, an imbalanced immunological barrier on the intestinal mucosa, and in an autoimmune disease [36]. It is decreased in children with sIgA deficiencies. The test was performed with the Secretory IgA ELISA Kit (ALPCO Diagnostics Cat. No. 30-8870).

### 

**Elastase:** The Elastase enzyme level can be used for the diagnosis or the exclusion of exocrine pancreatic insufficiency, which may be associated with chronic pancreatitis, cystic fibrosis, carcinoma of the pancreas, Diabetes mellitus Type 1, Shwachman-Diamond syndrome and other etiologies of pancreatic insufficiency. The test was performed with the Elastase ELISA BIOSERV Kit (Joli Medical Products Inc.).

**Short chain fatty acids (SCFA) **are the end products of anaerobic microbial fermentation of dietary fiber [37]. Levels thus reflect the concentration of intestinal flora as well as soluble fiber in the diet [38,39]. The SCFA distribution reflects the relative proportions of the beneficial SCFA (n-butyrate, propionate and acetate), thus providing an indirect measure of balance among the anaerobic organisms in the colon. These beneficial SCFA are crucial to the health of the intestine, serving as sources of fuel for the cells and the rest of the body. Decreased levels may reflect insufficient normal colonic flora, a diet low in soluble fiber, or prolonged intestinal transit time [39]. Abnormal level of short chain fatty acids in stool can indicate malabsorption and are used as metabolic markers. Levels of butyrate and Total SCFA's in mg/mL are important for assessing overall SCFA production, and are reflective of beneficial flora levels and/or fiber intake [40].

The "volatile" fatty acids in fecal samples were extracted into an HCl solution and quantified using a flame ionization detector (FID) following separation by gas chromatography(GC) [41,42]. The SCFAs that were measured include acetate, proprionate, butyrate, and valerate. Results were verified to be accurate and precise using quality control.

### Statistical Analysis

Several types of statistical analyses were carried out, depending on the research question being addressed. In comparing levels between groups (such as children with autism vs. typical children), 2-sided unpaired (independent sample) t-tests were used. The unpaired t-tests were either done assuming equal variance (if F-test results had p-values greater than 0.05), or assuming unequal variance (if F-test p-values were less than 0.05). For individual comparisons a p-value of 0.05 or lower was assumed significant. However, when multiple comparisons were considered, then a lower p-value was considered significant based on a Bonferroni analysis - this is defined at the beginning of each section of the results. Pearson correlation coefficients were obtained to determine the strengths of linear relationships among the variables involved in the analyses.

In some cases the data was not normally distributed. Those cases included lysozyme, lactoferrin, *E. coli*, and *Enterococcus*. For those cases, a non-parametric Wilcox analysis was used to compare the autism group and the typical group.

In addressing questions about the relationships between autism severity and GI severity, correlation analyses were performed.

## Results

### Beneficial Bacteria

Four types of beneficial bacteria were investigated, including bifidobacteria, *Lactobacillus *spp. (all strains, since all are beneficial), *E. Coli*, and *Enteroccus *- see Table 2. The children with autism had much lower levels of *Bifidobacterium *(-45%, p = 0.002), slightly lower levels of *Enterococcus *(-16%, p = 0.05 per Wilcox), and much higher levels of *Lactobacillus *(+100%, p = 0.00003).

**Table 2:** Beneficial bacteria from stool analysis.

	Autism/Aspergers	Control	*P*-value	% Difference
*Bifidobacterium*	1.6 ± 1.9	2.8 ± 1.8	0.002	-44%
*E.coli*	2.8 ± 1.7	2.4 ± 1.6	n.s.	
*Lactobacillus*	2.6 ± 1.4	1.3 ± 1.4	0.00002	+100%
*Enterococcus*	0.81 ± 1.4	0.97 ± 1.2	0.05 W	-16%

W - The data for the enterococcus was not normally distributed, so it was analyzed with a non-parametric Wilcox analysis.

Bacteriology culture values ranged from 0 to 4

### Dysbiotic Bacteria

Table 3 lists the dysbiotic bacteria, which were observed during aerobic culture growth. Since these bacteria were only observed rarely, the table lists how many individuals had measurable levels of these bacteria, and the average levels of the bacteria. There were no significant differences between the children with autism and the typical children.

**Table 3:** Dysbiotic bacteria found in stool analysis.

	Autism/Aspergers	Control	*P*-value
*Citrobacter youngae*	3.4% (0.14 ± 0.7)	5.1% (0.18 ± 0.8)	n.s.
*Citrobacter braakii*	None detected	2.6% (0.08 ± 0.5)	n.s.
*Proteus mirabills*	3.4% (0.14 ± 0.7)	2.6% (0.08 ± 0.5)	n.s.
*Salmonella*	1.7% (0.07 ± 0.5)	None detected	n.s.
*Citrobacter freundii*	1.7% (0.05 ± 0.4)	2.6% (0.18 ± 0.8)	n.s.

Values indicate percentage of participants with detectable (> 0) bacteria with average numerical values and standard deviation in parenthesis.

### Commensal Bacteria

Some bacteria are not believed to be especially beneficial or detrimental, and we term those commensal bacteria, and list the results for them in Table 4. Since these bacteria were only observed rarely, the table lists how many individuals had measurable levels of these bacteria, as well as the average level. The only possibly significant differences were that the autism group was more likely to have *Bacillus spp *(21% vs. 2.6%, p = 0.05) and less likely to have *Klebsiella oxytoca *(1.7% vs. 12.8%, p = 0.04).

**Table 4:** Commensal bacteria found in stool analysis.

	Autism/Aspergers	Control	*P*-value	% Difference
*Bacillus spp*.	21% (0.280 ± 64)	2.6% (0.05 ± 0.3)	0.05	+438%
*Klebsiella pneumonia*	12.1% (0.29 ± 0.94)	17.9% (0.23 ± 0.5)	n.s.	
Klebsiella oxytoca*	1.7% (0.03 ± 0.26)	12.8% (0.31 ± 0.9)	0.04	-89%
Pseudomonas aeruginosa*	10.3% (0.17 ± 0.6)	12.8% (0.21 ± 0.6)	n.s.	
*Haemolytic E.coli*	17.2% (0.64 ± 1.4)	20.5% (0.67 ± 1.4)	n.s.	
*Gamma strep*	29.3% (0.98 ± 1.6)	43.6% (0.79 ± 1.2)	n.s.	
*Alpha Haemolytic strep*	13.8% (0.40 ± 1.1)	46.2% (0.74 ± 1.0)	n.s.	
*Staphylococcus aureus*	13.8% (0.24 ± 0.7)	20.5% (0.28 ± 0.6)	n.s.	
*Enterobacter cloacae*	8.6% (0.09 ± 0.3)	12.8% (0.33 ± 1.0	0.07	-33%

*Indicates bacteria were either listed as commensal or dysbiotic depending on concentration.

The table indicates the percentage of participants with detectable (> 0) bacteria and in parenthesis average values and standard deviation. Numerical values of bacteriology culture ranged from 0-4.

### Yeast

The presence of yeast was determined by both culture and by microscopic observation. Yeast was only rarely observed by culture in the autism or typical groups, and the difference between the two groups was not significant, as shown in Table 5. Yeast was more commonly observed microscopically, but again the difference between the two groups was not significant.

**Table 5:** Cultured and microscopic yeast.

	Autism/Aspergers	Control	*P*-value
*Candida albicans*	15.6% (0.24 ± 0.7)	10.3% (0.13 ± 0.4)	n.s.
Other yeast	12.1% (0.12 ± 0.3)	2.6% (0.05 ± 0.3)	n.s.
Dysbiotic yeast	6.9% (0.14 ± 0.8)	None detected	n.s.
Yeast (microscopic)	70.7% (1.45 ± 1.4)	84.7% (1.87 ± 1.4)	n.s.

The table indicates the percentage of participants with detectable (> 0) bacteria and in parenthesis average values and standard deviation.

Numerical values of bacteriology culture ranged from 0-4. Variations in cultured and microscopic concentrations are typical due to the non-uniform distribution of yeast in the stool.

### Digestion

Elastase, a digestive enzyme, was measured and found to be similar in both the autism and typical groups, shown in Table 6. The presence of fat, muscle fibers, vegetable fibers, and monosaccharides were measured in both groups, and again no significant differences were found.

**Table 6:** Digestion/Absorption Markers.

	Autism/Aspergers	Control	*P*-value
Elastase (**μg/mL)**	487.0 ± 38.3	481.5 ± 61.1	n.s.
Fat Stain	0.17 ± 0.6	0.21 ± 0.6	n.s.
Muscle Fibers	0.19 ± 0.4	0.33 ± 0.5	n.s.
Vegetable Fibers	1.24 ± 0.6	1.28 ± 0.5	n.s.
Carbohydrates	0.17 +/- 0.68	None detected	n.s.

Fat stain, muscle fibers, and vegetable fibers were rated 0-4 with 0 = none, 1 = rare, 2 = few, 3 = moderate, 4 = many. Carbohydrates were listed as either negative or positive with a positive value indicating carbohydrate malabsorption from the presence of reducing substances in the stool sample.

### Inflammation

Possible markers of inflammation, including lysozyme, lactoferrin, white blood cells, and mucus, were investigated and shown in Table 7. The autism group had a lower level of lysozyme (-27%, p = 0.03 by Wilcox analysis), but no other significant differences.

**Table 7:** Summary of inflammatory markers found in the stool analysis.

	Autism/Aspergers	Control	*P*-value	% Difference
Lysozyme (ng/ml)	334 ± 282	464 ± 337	0.04	-28%
Lactoferrin (μg/mL	7.6 ± 18	4.4 ± 8.7	n.s.	
WBC	None detected	None detected	n.s.	
Mucus	1.7% Positive (0.02 ± 0.13)	None detected	n.s.	

Mucus was indicated as either negative or positive.

### Secretory IgA

Levels of secretory IgA were measured, and no significant difference between the two groups was observed as shown on Table 8. Secretory IgA was highly correlated with lysozyme for the autism group (R = 0.69, p < 0.001), and moderately correlated for the controls (R = 0.35, p < 0.01).

**Table 8:** Secretory IgA (sIgA) in stool (mg/dL)

	Autism/Aspergers	Control	*P*-value
Secretory IgA	166 ± 183	165 ± 249	n.s.

### Short Chain Fatty Acids

The presence of several short chain fatty acids (SCFA), including acetate, proprionate, butyrate, and valerate were measured. The total amount of SCFA was significantly lower in children with autism (-27%, p = 0.00003). Similarly, the levels of acetate, proprionate, and valerate were also lower in the autism group, as shown in Table 9. The lower level of SCFA's appears to be partly due to probiotic usage (see Effect of Probiotics section below). The low level of SCFA's is also partly due to lower sacchrolytic fermentation by beneficial bacteria, lower intake of soluble fiber, prolonged transit time due to constipation, and/or possibly increased absorption by the gut due to increased permeability. However, from this study we cannot determine among those possibilities.

**Table 9:** Summary of short chain fatty acids (SCFA's) in stool.

	Acetate	Butyrate	Propionate	Valerate	Total SCFA's
Autism/Asperger's	3.5 +/- 1.4	1.63 +/- 1.2	1.28 +/- 0.5	0.22 +/- 0.1	6.7 +/- 2.8

A-Probiotics (n = 19)	2.94 +/- 1.4	1.00 +/- 0.7	1.02 +/- 0.37	0.17 +/- 0.10	5.13 +/- 2.2

A-No-Probiotics (n = 38)	3.84 +/- 1.3	1.95 +/- 1.25	1.43 +/- 0.54	0.24 +/- 0.12	7.5 +/- 2.8

Controls	5.2 +/- 1.6	2.00 +/- 0.9	1.64 +/- 0.6	0.36 +/- 0.3	9.2 +/- 2.6

**Ttest Comparisons**					

A-Probiotics vs. A-No Probiotics	P = 0.02	P = 0.003	P = 0.004	P = 0.02	P = 0.002

A-Probiotics vs. Controls	P = 0.000002	P = 0.0001	P = 0.00007	P = 0.02	P = 0.0000002

A-No-Probiotics vs. Controls	P = 0.00009	n.s.	P = 0.09	P = 0.05	P = 0.006

All autism vs. Controls	P = 0.0000003 (-32%)	P = 0.005 (-18%)	P = 0.002 (-22%)	P = 0.005 (-39%)	P = 0.00002 (-27%)

Each fatty acid is expressed in units of mg/mL.

### Intestinal Health

The presence of RBC or occult blood was very rare in both groups, and not significantly different between the two groups. The fecal pH, a reflection of colonic pH, was very similar in both groups, although the autism group had a somewhat larger standard deviation, as listed in Table 10. (F-test for equal value p-value = 0.0004).

**Table 10:** Intestinal Health Markers.

	Autism/Aspergers	Control	*P*-value
RBC	5.2% (0.10 ± 0.55)	7.7% (0.08 ± 0.27)	n.s.
pH	6.46 ± 0.51	6.49 ± 0.29	n.s.
Occult Blood	3.4%	7.7%	n.s.

Red blood cells (RBC) reference range listed numerically with 0 = none, 1 = rare, 2 = few, 3 = moderate and 4 = many. Occult blood listed as percentage of cases with positive findings of occult blood.

### Gender Differences

Gender differences were investigated in the typical group for all of the measurements, and there were no statistically significant differences between males and females, expect for a possibly significant higher level of yeast in the females (p = 0.04). For the autism group, there were too few females to justify comparing the males vs. females.

### Effect of Probiotics

**Figure 1:**
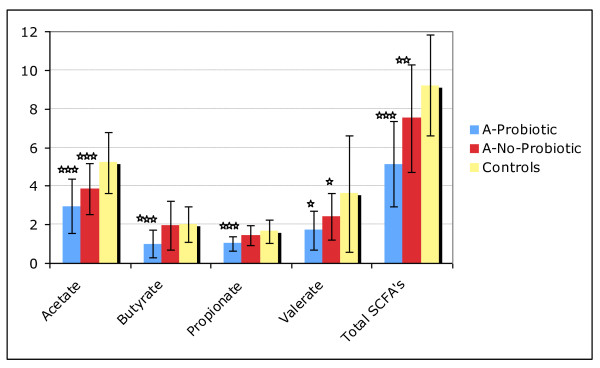
**Comparison of SCFA's for the Autism-Probiotic group, the Autism-No-Probiotic group, and the controls**. Note that the Valerate data is magnified 10x so that it is visible on the chart. The stars indicate the degree of significance of the result of a t-test comparison of the level of SCFA's in the autism subgroup (Probiotic or No-Probiotic) vs. the controls - * p < 0.05, ** p < 0.01, *** p < 0.001.

The autism group was split into two groups, A-Probiotic (those that used any type of probiotic daily) and those that did not use any probiotics, A-No-Probiotic. The only significant differences (p < 0.01) were that, compared to the A-No Probiotic group, the A-Probiotic group had a lower level of total SCFA's and each individual SCFA - see Table 9 and Figure 1. Probiotics did not have a significant effect on most of the beneficial bacteria, except for a marginally higher level of the level of lactobacillus in the A-Probiotic group compared to the A-No-Probiotic group (+30%, p = 0.08).

The A-Probiotic group had a lower level of lysozyme than did the A-No-Probiotic group, but the difference was not significant (277 +/- 231 vs. 371 +/- 302, n.s.). (see Lysozyme section). A t-test comparison of lysozyme in the autism groups with the Control group found a significantly lower level in the A-Probiotic group (31% lower, p = 0.03), but no significant difference for the A-No-Probiotic group (-11%, n.s.).

For the control group, only 5% used a probiotic, so they were not analyzed separately.

### Effect of Seafood and Fish Oil Consumption

The autism group was split into three groups based on consumption of seafood and fish oil (see Table 11):

**Table 11:** Consumption of Fish and Fish Oil.

	Lactobacillus	pH
A-Fish (n = 7)	0.71 +/- 0.95	6.20 +/- 0.25

A-Fish Oil (n = 15)	3.00 +/- 1.26	6.84 +/- 0.52

A-No Fish (n = 34)	2.90 +/- 1.37	6.44 +/- 0.50

C-Fish (n = 13)	0.85 +/- 1.1	6.47 +/- 0.24

C- No Fish (n = 22)	1.60 +/- 1.5	6.52 +/- 0.31

		

**T-Test Comparisons**		

t-test: A-Fish vs. A-Fish Oil	p = 0.0009	p = 0.009

t-test: A-Fish vs. A-No Fish	p = 0.0007	n.s.

t-test: A-Fish Oil vs. A-No Fish	n.s.	p = 0.05

		

t-test: C-Fish vs. C-No Fish	n.s.	n.s.

Autism Subgroups and Control Subgroups compared. Only biomarkers with p < 0.01 are listed

A- Fish - Consumption of seafood more than 2x/month - (57% also consumed fish oil daily)

A- Fish Oil - No consumption of seafood; consumes fish oil supplement daily

A-No Fish - No consumption of seafood or fish oil

The typical group was divided similarly, but none of the typicals were consuming fish oil regularly.

A t-test comparison of those groups found only two biomarkers which differed between the groups with p < 0.01, namely lactobacillus and pH. Regarding Lactobacillus, the A-Fish Oil and the A-No Fish had similar levels, but the A-Fish had dramatically lower levels than the other two groups. Regarding pH, the A-Fish Oil group had slightly higher pH values than the other two groups.

The control group was split into only two groups, C-Fish and C-No Fish, because none of the controls consumed fish oil (see Table 11). There were no significant differences between the two groups.

### Kruskall-Wallis Analysis on Lactobacillus

Since exploratory data analysis with t-tests found that levels of lactobacillus were associated with both fish consumption and probiotic usage, a Kruskall-Wallis rank sum test (which treat the data as the ranked values that they are) was conducted. For the autistic group, Lactobacillus levels were very significantly related to levels of fish consumption (p = 0.0008) and adding other variables (ie, fish oil consumption or probiotics) did not add to the significance of the relationship. No relationships were found for the control group.

### Effect of Gut Symptoms in Autism Group

The autism group had significantly greater gut symptoms than the control group in part because the control group was chosen to be "in good health: no stomach/gut problems." To investigate the effect of gastrointestinal problems on the results, the autism group was split into two groups: Low-GI-Problems (defined as 6-GSI of 3 or less) and High-GI-Problems (defined as 6-GSI score above 3). The two groups were compared for all the biomarkers reported above, and there were no significant differences (p < 0.01) in any biomarkers.

**Figure 2:**
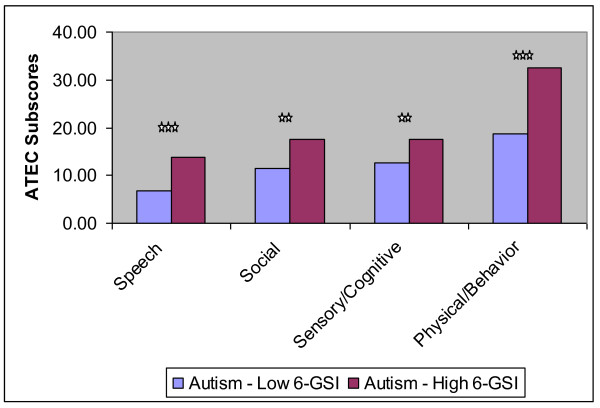
Comparison of ATEC subscores for the Autism-Low 6-GSI group (few GI problems) and the Autism High 6-GSI group (many GI problems); the stars indicate the significance (*** p < 0.001, ** p < 0.01).

However, the ATEC scores of the two groups were very different, and this is reported in Table 12 and displayed in Figure 2. The total ATEC score was 66% higher in the High-GI-Problem group (p = 0.00002), and the four subscales were also higher (+40% to +103%, p < 0.01).

**Table 12:** ATEC scores for the Autism-Low-GI-Problem group (6-GSI score of 3 or lower) and for the Autism-High-GI-Problem group (GSI-6 score above 3).

	Autism-Low-GI-Problem Group (n = 22)	Autism-High-GI-Problem Group (n = 34)	% difference	p-value from ttest
6-GSI score	1.4 +/- 0.8	5.4 +/- 1.7	+295%	

ATEC-total	49.0 +/- 21	81.5 +/-27.6	+66%	0.00002

**ATEC Subscales**				

Speech/Language/Communication	6.7 +/- 4.4	13.7 +/- 8.0	+103%	0.0005

Sociability	11.5 +/- 5.3	17.6 +/- 7.6	+53%	0.002

Sensory/Cognitive Awareness	12.6 +/- 7.0	17.6 +/- 6.8	+40%	0.01

Health/Physical Behavior	18.7 +/- 9.1	32.6 +/- 12.0	+74%	0.00003

### Correlations with Gut Symptoms and Autism Severity

**Figure 3:**
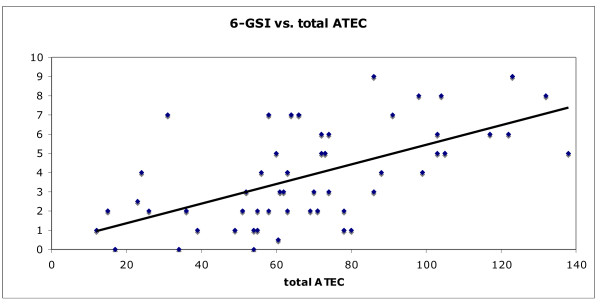
**6-Item Gastrointestinal Severity Index (6-GSI) vs. total ATEC score**.

For the autism group, the 6-GSI was found to strongly and very significantly correlate with the total ATEC (r = 0.60, p < 0.001); see Figure 3. Due to this strong correlation, correlations with the ATEC subscales were also determined and are listed in Table 13.

**Table 13:** Correlations between total ATEC and subset divisions and total GI Severity.

	Autism/Aspergers	*P*-value
Total ATEC	*r*= 0.60	< 0.001
I. Speech/Language/Communication	*r = *0.47	< 0.001
II. Sociability	*r = *0.50	< 0.001
III. Sensory/Cognitive Awareness	*r = *0.34	0.01
IV. Health/Physical/Behavior	*r *= 0.62	< 0.001

Correlations of each biomarker of gut status with the 6-GSI and the total ATEC scores were investigated. (However, comparisons with individual bacteria and yeast were not done, since in most samples they were not detected, and the large number of the comparisons made the required per-comparison p-value very low). For the 6-GSI there was a small correlation with carbohydrates (R = 0.27, p = 0.04). For the total ATEC, there was a small correlation with lysozyme (R = 0.29, p = 0.03). However, since multiple correlation analyses were conducted, the cut-off for significance (p = 0.05) should be divided by the number of tests. So, these correlations are at most only possibly significant, and a larger study would be needed to assess this possible weak correlation.

## Discussion

The very strong correlation of the 6-GSI with the ATEC and its subscales indicates that there is a very strong association of gastrointestinal symptoms and autistic symptoms. Of course, association does not mean causation, but the effectiveness of oral, non-absorbable antibiotics in temporarily reducing autistic symptoms [15] does suggest that the relationship may be causal; ie, we hypothesize that gastrointestinal problems may significantly contribute to autistic symptoms in some children.

The lower levels of SCFA's were extremely significant. The A-Probiotic group had much lower levels than the controls (-44%, p = 0.0000002), but the A-No-Probiotic group also had lower levels that the controls (-19%, p = 0.006). This suggests that there are either lower amounts of beneficial bacteria which produce SCFA's, a lower intake of soluble fiber, a longer transit time, and/or increased absorption due to increased gut permeability. The latter possible explanation is very intriguing because of work by MacFabe *et al. *2007 [17], which demonstrates that SCFA's can induce autistic-like symptoms when injected into rats. In other words, if lower levels of SCFA's in the stool are due to increased absorption, then this presumably would lead to higher level of SCFA's entering the bloodstream, and hence would exacerbate autistic symptoms. If however, lower levels of SCFA's in the stool are due to lower amounts of SCFA-producing bacteria or low fiber intake, there may not be higher SCFA levels in the bloodstream. So, our results suggest that measurements of SCFA's in blood and/or urine samples are warranted.

Lysozyme is an important part of the immune system, and protects the gut from pathogenic bacteria by enzymatic attack of their cell walls. It is secreted by recruited macrophages, monocytes, and granulocytes at the site of inflammation. Infants fed formula without lysozyme have three times the rate of diarrheal disease [43]. In this study, lysozyme levels were lower in children with autism (-28%, p = 0.04); this difference was significant for the A-Probiotic group, but not for the A-No-Probiotic group. We hypothesize that probiotics provide some limited defense against pathogenic bacteria, and thus decrease the need for the immune system to excrete lysozyme.

The unusually broad distribution of pH in the autistic group suggests that there is a general disregulation of pH, which could affect digestion and bacteria. pH was negatively correlated with lysozyme (R = -0.34, p = 0.01), suggesting that higher pH is associated with lower levels of lysozyme and vice versa. Also, pH was even more strongly negatively correlated with total SCFA (R = - 0.44, p < 0.001), presumably because SCFA's contribute to colonic pH.

The lower amounts of bifidobacteria (-44%, p = 0.002) in children with autism is consistent with a pyrosequencing study [14] that also found lower levels of bifidobacteria (-37%, p = 0.05) in children with autism, and suggests that supplementation with bifidobacteria is worth investigating. The high levels of lactobacillus in the autism group was partly associated with decreased rate of seafood consumption.

It was interesting that dysbiotic bacteria were present at similar (low) levels in both groups. However, it should be noted that many bacteria such as clostridia would not be detected by the culture-based methods used in the present study, except for *Clostridium difficile *if present at high levels. Feingold's pyrosequencing study [14] estimated that typical children normally have about 500-600 species of bacteria in their gut and estimated that children with autism have about twice that, so it is important to remember that we are only able to culture a small fraction of the species present.

The finding that yeast levels were similar in both the autistic and control group is interesting, as there has been a great deal of speculation that yeast infections are a major problem in autism. Our data indicates that yeast is present at normal levels in the stool of this group of children with autism. A study by Horvath and Perman [21] reported that 43% of children with autism undergoing endoscopies had a positive fungal culture for yeast in their duodenal juice, vs. 23% of age-matched controls with other gastrointestinal problems requiring endoscopies. Since their study involved children with severe enough symptoms to warrant endoscopies, the greater symptom severity may explain some of the difference with our study. Since the survey by the Autism Research Institute of over 25,000 parents' reports that parents find antifungals to be one of the most effective medications for improving behavior [44], our findings are puzzling. It is possible that children with autism are more sensitive to even a normal level of yeast. Also, it is possible that antifungals have other effects, such as reducing inflammation.

## Study Limitations

1) The diagnosis of an autism spectrum disorder was made by a qualified medical professional prior to enrollment in the study, but there was no additional verification. Similarly, for the neurotypical children, no additional verification was made beyond the parental report.

2) One limitation of this study is that the children with autism had more gastrointestinal problems, lower fish consumption, more fish oil consumption, and more probiotic usage. This greatly complicated the study analysis, but provided interesting insights into these effects. The results for these subgroups should be interpreted cautiously, if at all, due to the smaller size of the subgroups and potential confounding of fish and fish oil consumption results. Larger, more carefully designed studies are needed to investigate these issues properly.

## Conclusion

The strong correlation of the 6-GSI and the ATEC (r = 0.59) strongly suggests that gastrointestinal problems are associated with autism severity. The autism group with low GI problems had much lower scores on the ATEC and each of its four subscales compared to the the autism group with high GI problems.

The autism group had a low level of SCFA's, partly associated with probiotic usage, and probably partly due to either lower sacchrolytic fermentation by beneficial bacteria, due to their relative absence, lower intake of soluble fiber, longer transit time, and/or increased gut permeability.

The lower levels of *Bifidobacterium *and higher levels of *Lactobacillus *suggests an imbalance in beneficial bacteria. Increased levels of *Lactobacillus *were strongly associated with decreased seafood consumption is the autism group (p = 0.0008).

Lysozyme were lower in the autism group compared to controls, and the lower levels of lyszoymes were associated with probiotic usage; it is possible that probiotics decrease the need for lysozyme to be excreted to defend against pathogenic bacteria.

## Competing interests

The authors declare that they have no competing interests.

## Authors' contributions

JBA was the principal investigator, oversaw the study design, conducted most of the data analysis, and wrote most of the paper. LJJ assisted with participant recruitment, data analysis, and paper preparation. LDP assisted with participant recruitment and data analysis. DQ oversaw the testing at Doctors Data, led the writing of the laboratory methodology sections, and helped with interpreting the results. RAR assisted with statistical analysis and editing the paper. All authors read and approved the final version of the paper.

## Pre-publication history

The pre-publication history for this paper can be accessed here:

http://www.biomedcentral.com/1471-230X/11/22/prepub
